# Biomechanical modelling of the pelvic system: improving the accuracy of the location of neoplasms in MRI-TRUS fusion prostate biopsy

**DOI:** 10.1186/s12885-022-09432-4

**Published:** 2022-03-28

**Authors:** Muhammad Qasim, Dolors Puigjaner, Joan Herrero, Josep M. López, Carme Olivé, Gerard Fortuny, Josep Garcia-Bennett

**Affiliations:** 1 Departament d’Enginyeria Informàtica i Matemàtiques, Universitat Rovira i Virgili, Tarragona, Catalunya Spain; 2 Departament d’Enginyeria Química, Universitat Rovira i Virgili, Tarragona, Catalunya Spain; 3 Servei de Radiologia, Hospital Universitari de Bellvitge, Barcelona, Catalunya Spain

**Keywords:** Magnetic resonance imaging (MRI), Prostate neoplasm, Code-Aster, Transrectal ultrasound (TRUS), TRUS-guided biopsy

## Abstract

**Background:**

An accurate knowledge of the relocation of prostate neoplasms during biopsy is of great importance to reduce the number of false negative results. Prostate neoplasms are visible in magnetic resonance images (MRI) but it is difficult for the practitioner to locate them at the time of performing a transrectal ultrasound (TRUS) guided biopsy. In this study, we present a new methodology, based on simulation, that predicts both prostate deformation and lesion migration during the biopsy.

**Methods:**

A three-dimensional (3-D) anatomy model of the pelvic region, based on medical images, is constructed. A finite element (FE) numerical simulation of the organs motion and deformation as a result of the pressure exerted by the TRUS probe is carried out using the Code-Aster open-source computer software. Initial positions of potential prostate lesions prior to biopsy are taken into consideration and the final location of each lesion is targeted in the FE simulation output.

**Results:**

Our 3-D FE simulations show that the effect of the pressure exerted by the TRUS probe is twofold as the prostate experiences both a motion and a deformation of its original shape. We targeted the relocation of five small prostate lesions when the TRUS probe exerts a force of 30 N on the rectum inner wall. The distance travelled by these lesions ranged between 5.6 and 13.9 mm.

**Conclusions:**

Our new methodology can help to predict the location of neoplasms during a prostate biopsy but further studies are needed to validate our results. Moreover, the new methodology is completely developed on open-source software, which means that its implementation would be affordable to all healthcare providers.

## Background

Cancer is a great burden on society and Prostate Cancer (PCa) is the tumor with the highest incidence and is the third cause of mortality from cancer in men in the EU [1]. In 2018, approximately 1.3 million new PCa cases were registered (representing around 7.1% of total cancer cases) and almost 359,000 deaths were caused by PCa all around the world [2, 3]. PCa typically arises in the peripheral zone, which is near the rectum wall. Transrectal ultrasound (TRUS) biopsies are commonly used in clinical practice due to their safety and efficiency [4]. In the last few years there has been a tendency to include information from magnetic resonance images (MRI) to guide the prostate biopsy process. As MRI becomes more sensitive in detecting small lesions, the sampling of these small lesions becomes increasingly dependent upon the targeting accuracy of the practitioner [5]. State-of-the-art MRI-TRUS fusion platforms rely on the procedure known as registration, which consists of the superposition of the MRI image set with the corresponding live TRUS images [6].

Two methods of MRI/TRUS fusion registration have been developed, a rigid and an elastic registration. The first involves superimposing the MRI images onto the TRUS images after paired landmarks are established in both. The second method, elastic registration, applies statistical segmentations of the prostate and algorithms to deform the MRI images according to the TRUS deformation [7]. Both methods have shown a significant reduction of false negative results (14% for rigid [8] and 31.4% in elastic [9] compared to TRUS alone). However, rigid registration does not take into account the pressure of the TRUS probe on the rectum wall which results in motions and deformations of the prostate and surrounding pelvic organs. Consequently, multiple biopsy samples are required in order to target one single lesion [10], increasing undesirable associated complications. The elastic registration method assumes that the prostate is deformed homogenously throughout its different zones and ignores the effect that periprostatic structures can have on the deformation. The biopsy accuracy of both rigid and elastic deformation has been reported to be under 3 mm [11, 12]. A study carried out with a phantom model found no significant differences in registration errors between rigid and elastic registration (4.11 vs. 4.87 mm, *p* = 0.05) [13], although a slightly higher cancer detection rate has been reported with elastic compared to rigid registration [14].

In this study we present an alternative methodology, based on a simulation of the pelvic region, that can improve the location accuracy of prostate neoplasms during an MRI-TRUS fusion biopsy. Our methodology is entirely based on open-source software and therefore can be implemented at a comparatively low cost. The main goals of this study are:

- To integrate an accurate and realistic 3-D geometry of the pelvic region and the constitutive properties of the tissues involved into a computational finite element (FE) model.
- To use this FE model to simulate the prostate’s biomechanical response to the pressure applied by the TRUS probe on the rectum wall during a TRUS guided biopsy.
- To predict the actual location of prostate neoplasms during a TRUS guided biopsy.

## Methods

**Fig. 1:**
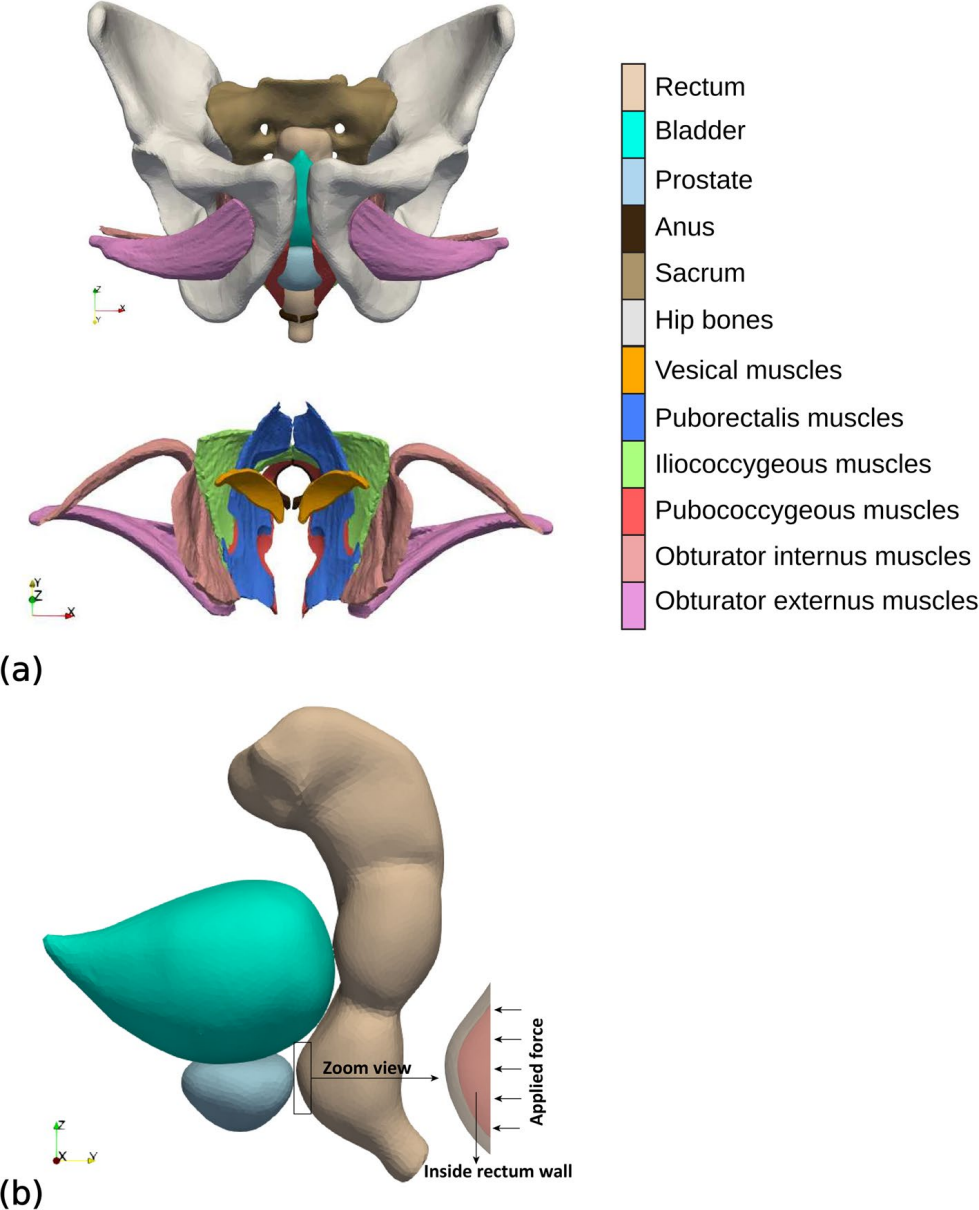
**a** Transverse view of the geometry model for the pelvic region. For the sake of clarity only the involved muscles are included in the bottom plot. Note that in the frontal body view the *X*-axis points to the right, the *Y*-axis points toward the dorsal region and the *Z*-axis points upwards (into the cranial region). **b** Lateral view of the rectum, bladder and prostate organs with a magnification of the interior region of the rectum where the TRUS probe exerts pressure

The geometry model of the male pelvic region used in the current simulation study is shown in Fig. 1. It is a realistic model that includes pelvic bones (hip and sacrum bones), pelvic muscles (obturator internus, obturator externus, iliococcygeus, pubococcygeus, puborectalis and vesical muscles), anus, rectum, bladder and the prostate transitional zone (TZ) and peripheral zone (PZ). Our geometry model is based on computerized tomography (CT) images available in the BodyParts3D database for anatomy [15]. This dictionary-type anatomical database provided (3-D) triangular surface models for each of the individual elements (organs, muscles, bones) involved in our male pelvic model. These surface models were conveniently refined and modified (undesirable intersections of adjacent elements were removed) using home-made and open source [16] software to obtain physically consistent computational meshes. The consistent surface meshes, defined by a total of 180,766 triangles, were then uploaded into the Gmsh open-source software [17] where 3-D tetrahedral volume meshes were obtained and optimized. The ensemble of volume meshes, one for each individual element in the geometry model, were finally compounded into a single computational mesh consisting of 655,355 tetrahedra.

Numerical simulations were performed using the Code-Aster open-source FE software [18]. In these simulations we replicated as closely as possible the real conditions of the clinical practice of a TRUS guided biopsy. We assumed that a force of 30 N was orthogonally exerted onto a surface patch of the rectum wall in an area of 258 mm^2^ (a sketch can be seen in the inset of Fig. 1b). The force is an estimate of the one applied by the practitioner during TRUS. The area is representative of the average shape and size of the contact between the transducer (EC9–4 Siemens Acuson sidefire endocavitary probe) and the rectum wall. In addition, we assumed that the sacrum and hip bones would be immobile during the biopsy and thus we set zero deformation boundary conditions for these bones.

We assumed an isotropic linear elastic behavior for all the tissues included in our model. According to this behavior, the relative deformation of the material (strain) is proportional to the force per unit area (stress) applied to it. To describe a linear elastic behavior two parameters are needed: the Young’s modulus, *E*, which measures the stiffness of the material (technically, *E* is the ratio between stress and strain so that the larger *E*, the stiffer the material) and the Poisson’s ratio, η, which measures the relative volume change [19]. In the present study, the Young’s modulus for the TZ and PZ tissue were chosen as those obtained from shear wave elastography by Wang et al. [20] for their patient case 6. Following Krouskop et al. [21], the Poisson ratio (η) for prostate tissues was set to 0.495. The material properties for all the other involved tissues were also obtained from the literature [22–27] and their specific values are summarized in Table 1. The main outputs of a FE simulation are the deformation and stress fields. In the present study, we focused on the calculated deformation field.

**Table 1:** Material properties of the elements in our model for the pelvic region: Elastic modulus (*E*), Poisson’s ratio (η) and density (ρ)

Organ	*E* (kPa)	η	ρ (kg/m^3^)	Source
Prostate transitional zone	43	0.495	1500	[20]
Prostate peripheral zone	18	0.495	1500	[20]
Bladder	10	0.499	1500	[22]
Rectum	5180	0.499	1500	[23]
Anus	10	0.499	1500	[24, 25]
Obturator internus muscles	15	0.4	1500	[24, 25]
Obturator externus muscles	15	0.4	1500	[24, 25]
Iliococcygeous muscles	15	0.4	1500	[24, 25]
Pubococcygeous muscles	15	0.4	1500	[24, 25]
Puborectalis muscles	15	0.4	1500	[24, 25]
Vesical muscles	150	0.4	1000	[26]
Sacrum bones	11 × 10^6^	0.26	1640	[27]
Hip bones	11 × 10^6^	0.26	1640	[27]

To simulate the displacement of very small lesions within the prostate, which are the most difficult to register with the fusion procedure, several mesh nodes were selected in the original geometry and their displacements were tracked by measuring their resulting location in the deformed geometry.

## Results

**Fig. 2:**
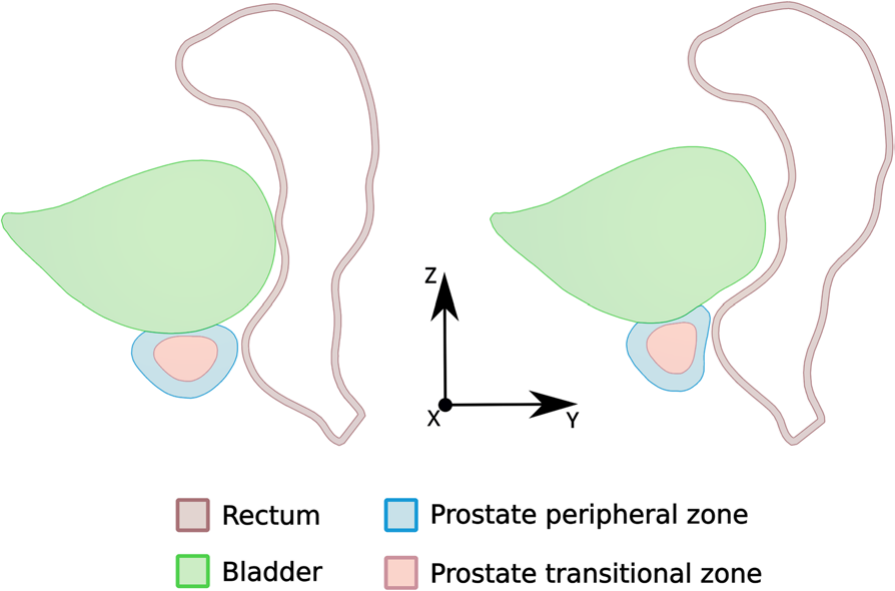
Profiles of the prostate transitional zone (TZ), prostate peripheral zone (PZ), bladder and rectum in the medial (*X* = 0) plane: original (left) and deformed (right) geometries

**Fig. 3:**
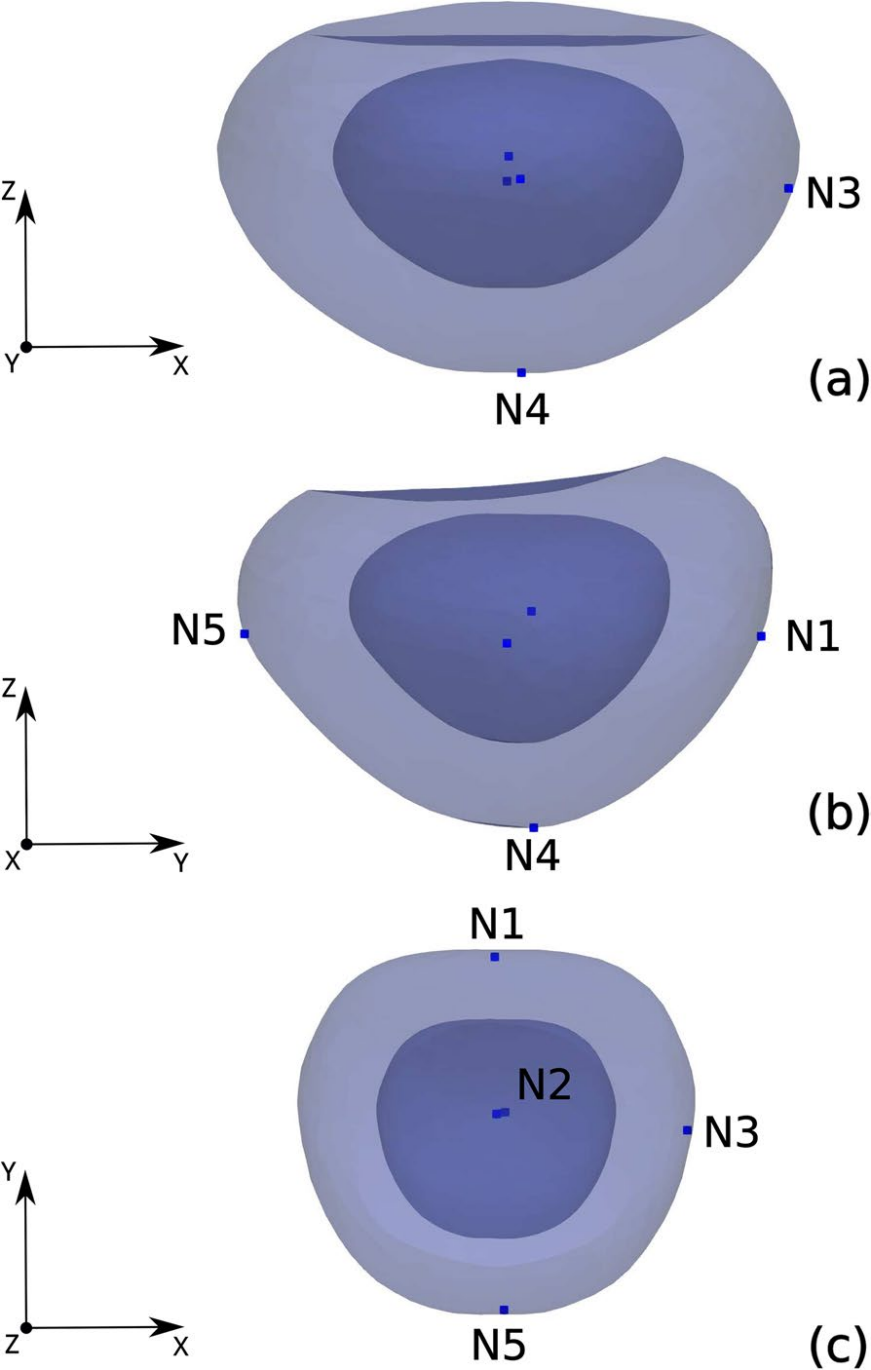
Location of the five selected nodes (potential neoplasms) in the prostate. **a** Coronal (posterior) view. **b** Sagittal view. **c** Axial view

**Fig. 4:**
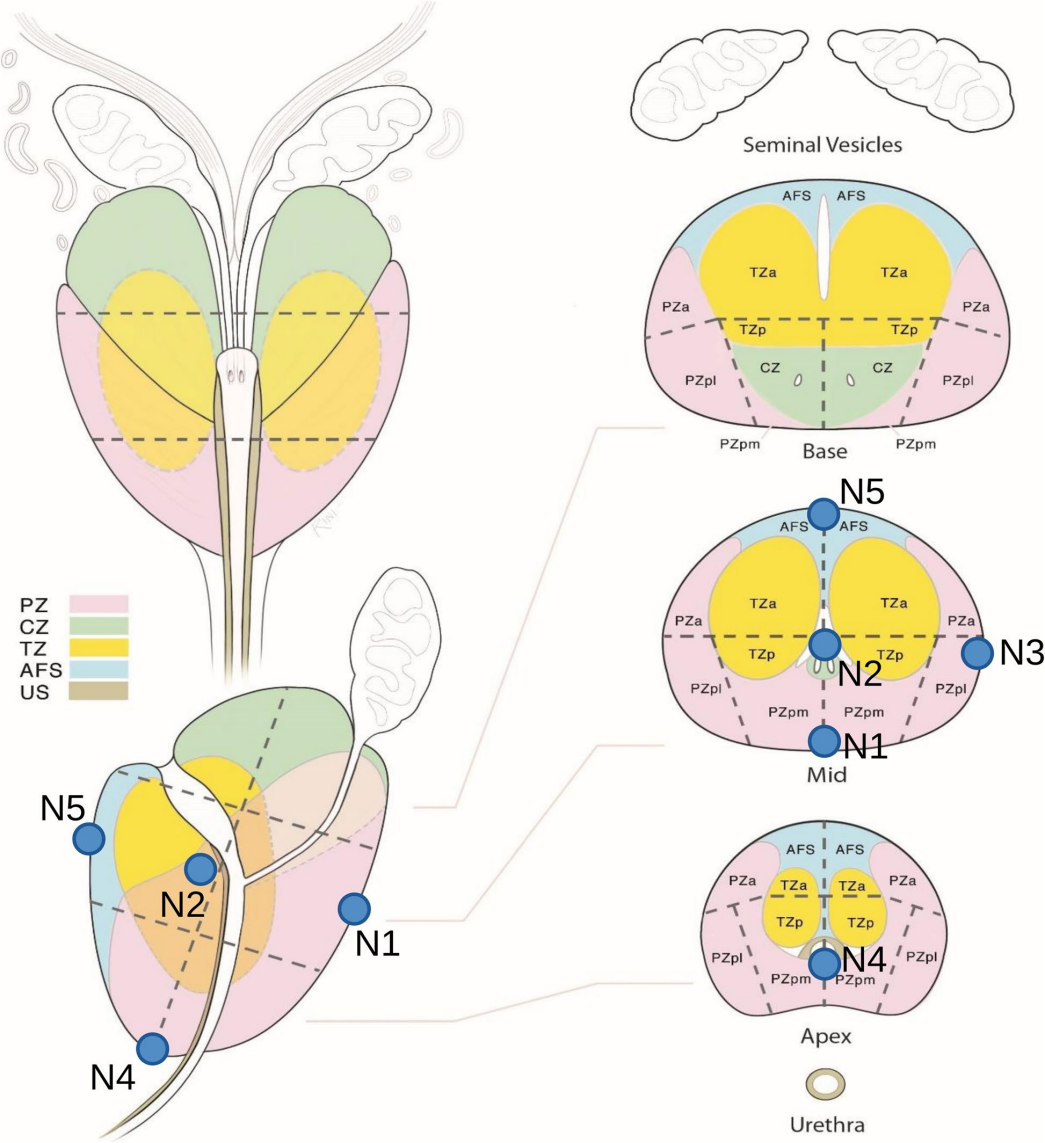
Approximate situation of the five selected nodes in the PI-RADS maps [28] as published by the American College of Radiology [29] under a creative commons (CC BY-NC-ND 4.0) license [30]. We have modified the original maps image by superimposing the symbols (circles) and labels denoting the situation of each node

Figure 2 shows the predicted deformations experienced by the rectum, bladder and prostate when the TRUS probe exerts a force of 30 N on the rectum inner wall. The region where the probe exerts the force, that is, the anterior part of the rectum and the posterior part of the prostate (see Fig. 1a), is that which experiences the highest deformation. The deformed rectum pushes against the prostate, displacing it towards the ventral region, together with a significant non-uniform deformation of the TZ and PZ geometry. The maximum displacements along the *Y* direction for the rectum, TZ and PZ contours in the plane of Fig. 2 were respectively 13.5, 11 and 12 mm. At the same time, the bladder region in contact with the prostate was deformed and cranially displaced (*Z* direction) approximately 9 mm. Note that the high difference in stiffness of bones and muscles contributes to the generation of non-uniform deformations of the rectum, bladder and prostate. To simulate the displacement of small lesions in the prostate we selected five different nodes, as defined in the two leftmost columns of Table 2 and sketched in Figs. 3 and 4. In relation to the zones defined by the PI-RADS maps [28, 29], nodes N1 and N4 are located near the outer surface of PZpm, N3 and N5 are located near the PZpl and AFS outer surfaces, respectively, and the N2 node is within the TZ.

**Table 2:** Coordinate displacements and their magnitudes of prostate lesions with respect to nodes after applying TRUS pressure at rectum wall

Node	Location in prostate	Displacements (mm)			
		*DX*	*DY*	*DZ*	Magnitude
N1	PZpm, mid level	0.94	−12.91	5.16	13.93
N2	TZp, mid level	−0.66	−7.84	2.52	8.26
N3	PZpl, mid level	3.37	−4.56	1.70	5.20
N4	PZpm, apex level	0.68	−6.56	1.56	6.78
N5	AFS, mid level	0.34	−5.53	0.94	5.61

**Fig. 5:**
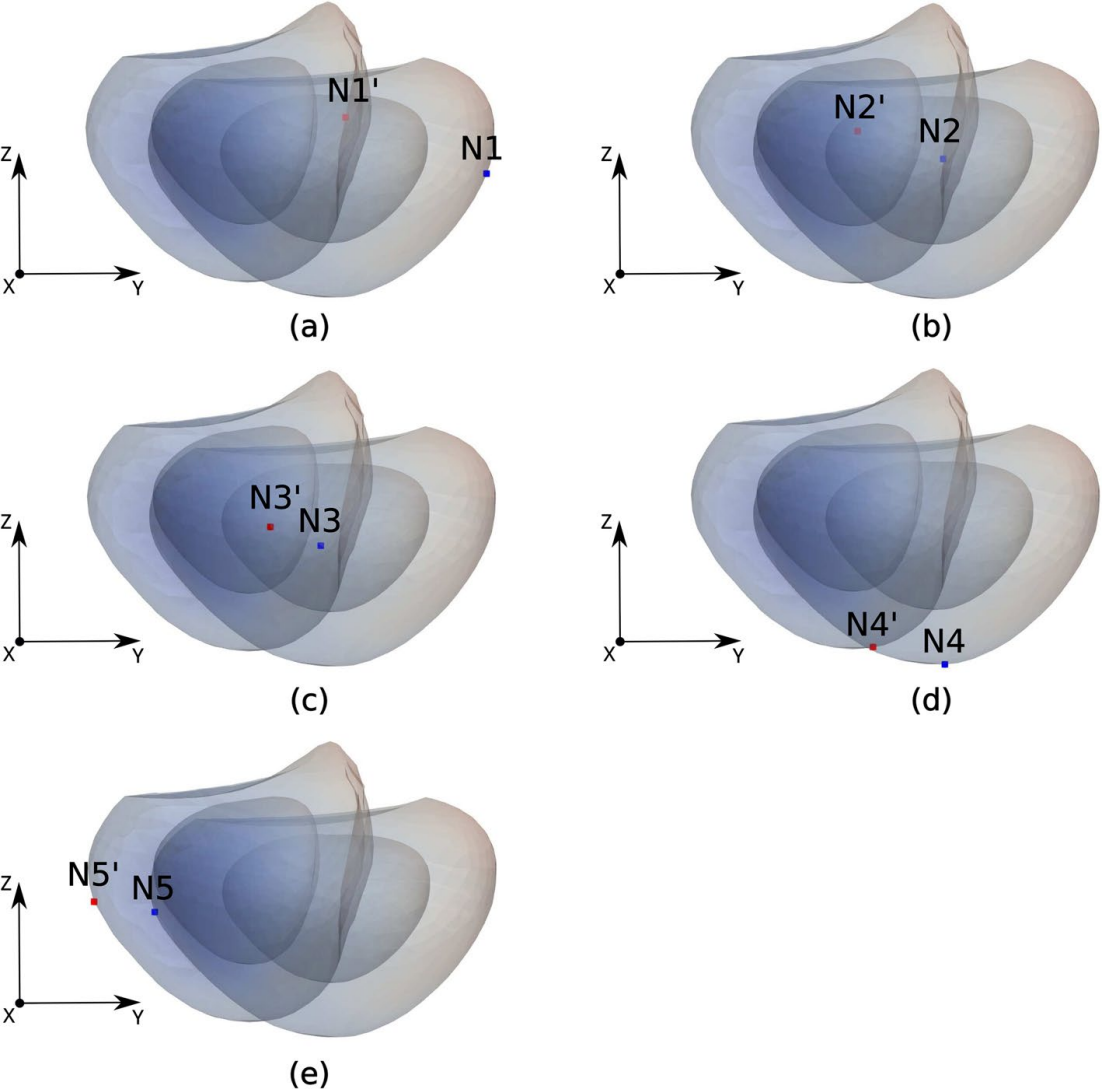
Each plot shows the projection of the 3-D original and deformed prostate surfaces into the sagittal plane. Blue and red squares respectively denote the initial and final locations of each of the five selected nodes: **a** N1, **b** N2, **c** N3, **d** N4 and **e** N5. Note that for the sake of clarity final locations are denoted with a prime added to the node label

Figure 5 shows superimposed projections in the sagittal plane of the original prostate geometry and the deformed geometry when the TRUS probe exerts a force of 30 N. The comparison of the original and deformed surface contours reveals the two main effects of the TRUS probe pressure: a motion (a displacement in the absolute frame of reference) and a deformation (a change in volume and shape) of the prostate gland. The original prostate shape in Fig. 5 still recalls what would be the surface of an idealized ellipsoid, whereas the deformed contour features a far more irregular shape. The displacements of the selected nodes are also portrayed in Fig. 5. The detailed information of node displacements along each of the 3-D coordinate-axes, as well as its magnitude, are presented in Table 2. The distance travelled by the nodes is between 5.20 and 13.91 mm, with the N1 node experiencing by far the highest displacement.

**Fig. 6:**
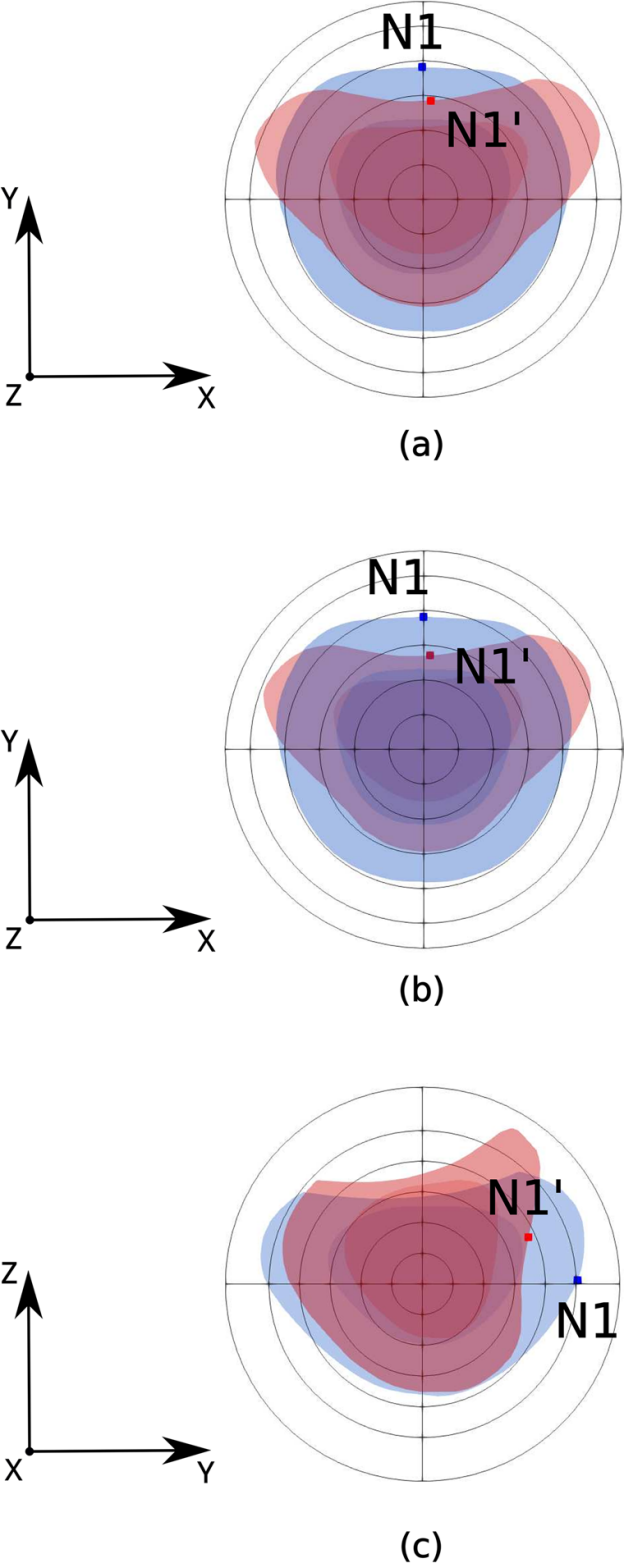
Superposition of original (blue) and deformed prostate in polar coordinates. **a** Axial views with *Z* = 0 for both the original and the deformed prostate. **b** Axial views taken at the plane where N1 (N1’) is located for the original (deformed) prostate. **c** Sagittal views taken at the plane where N1 (N1’) is located for the original (deformed) prostate

In Fig. 6a we present an axial slice keeping the same vertical location (*Z* = 0) where the N1 lesion was observed in the original MRI images. It can be clearly seen in this figure that, even after rigid registration (superimposition of slices), the large deformation experienced by the prostate makes the final slice quite different from the initial one. At first sight, however, it seems that given the initial location of N1 in the axial plane, its final location (N1’) would not be difficult to estimate. In this respect, the N1 node appears to be rather favourably placed, as it is very close to the prostate external wall and lies in the midsagittal (*X* = 0) plane. Note that in Fig. 6a we have not plotted the real N1’ 3-D location but its projection in the axial plane. Our biomechanical model predicts a small leftwards displacement of N1 as a result of the deformation, which is consistent with the fact that a real human body will never be 100% symmetric. The sagittal slices superimposed in Fig. 6c show that the biggest source of error in the final N1’ location, when sought in the original (*X* = 0) axial plane, is in the normal coordinate (*Z*). Our biomechanical FE simulation predicts that the final N1’ location is not in the original axial location (*Z* = 0) but in the plane with *Z* = 5.16 mm (see Table 2). This is taken into account in Fig. 6b, where registration is performed using the proper slice for N1’. Note that both axial polar plots in Fig. 6a and b are quite similar. Thus, on the one hand, in the example considered here (for the N1 node) the practitioner would have been able to estimate fairly well the *X* and *Y* coordinates of the lesion in the TRUS image, regardless of the particular axial plane being visualized. However, as clearly shown is Fig. 6c, even with an accurate projection of the lesion in the axial plane a large error could be made in the estimation of its axial location (*Z*).

## Discussion

Registration is aimed to help the practitioner locate a neoplasm by removing (or at least greatly reducing) one of the consequences of TRUS, prostate motion and deformation (see Fig. 5). Ideally, the two superimposed slices (from MRI and TRUS) should show similar shapes of the prostate in order to target a lesion with a reasonable degree of accuracy. Our results, obtained from numerical simulations, revealed that a 30 N force exerted by the TRUS probe on the rectum wall led to a significant deformation of the TZ and PZ. From a radiological standpoint, it is important that lesions located in the posteromedial PZ (N1) and in the TZ (N2) of the mid-gland are those experiencing the largest displacements. This fact is not surprising, considering that N1 is the node located closer to the rectum, where it is most directly affected by the probe pressure. On the other hand, the lowest displacement of the N3 node may be attributed to the restraint imposed by the puborectalis and pubococcygeus muscles. Note that the deformation induced by the probe also implies a strong departure from symmetry with respect to the midcoronal plane. Lesions located laterally (N3), anteriorly (N5) or in the apex level (N4) experienced the smallest displacements. These lesions tend to be tracked by MRI-TRUS fusion methods as they are difficult to reach by systematic biopsies.

Following the approach proposed by Igarashi et al. [31], Fig. 6 shows superimposed slices of the original and deformed prostate with the different locations of node N1. Figure 6 intends to approximate the type of representation that a MRI-TRUS rigid registration procedure would generate when intending to track the N1 node. The idea behind the polar coordinate framework is to determine the origin of the polar system in the original (undeformed) slice, the corresponding origin in the deformed slice and then to apply a translation of the latter origin into the former one, resulting in the superimposition of both images. The example shown in Fig. 6 illustrates the difficulty in estimating the final neoplasm.

In this study we have restricted our FE simulations to the tracking of very small prostate lesions, which can be assimilated to a node in the computational mesh. The present methodology could, however, be easily extended to track the deformation and displacement of larger lesions (neoplasms), by defining a group of volume elements for each neoplasm and assigning a different set of properties (*E*, η) for the tumoral tissue.

Several previous studies aimed to improve the location accuracy of prostate neoplasms during a TRUS guided biopsy. Different perspectives were applied in these studies. Some authors [20, 32, 33] proposed statistical and biomechanical methods to investigate the prostate deformation under different ultrasound probe insertion conditions. In particular, Wang et al. [20] provided patient-specific biomechanical parameters, acquired from ultrasound elastography, for the prostate transitional (TZ) and peripheral (PZ) zones in a data set with twelve patients. Other authors [32, 33] used finite element (FE) based statistical motion models (SMM) to estimate the shape adopted by a prostate when it was deformed due to TRUS probe pressure. Baratha et al. [34] proposed a deformable image registration system based on a biomechanical 3-D FE modelling with linear elastic properties for the prostate. Marchal et al. [35] implemented a discrete modelling method to simulate the displacement and deformation of the prostate due to both internal interactions between organs and external interactions between organs and surgical tools, such as the needle. Other studies [36–38], performed in the context of prostate radiotherapy, provided also interesting information on how to address the challenging problem of motion and deformation of prostate.

A recent trend in the image registration field is the development of AI methodologies based on deep neural networks [5, 31, 39, 40]. One crucial issue in these methodologies is the definition of robust strategies that generate the samples used in the network training step [5]. It is quite common to build these samples by taking image pairs that were registered manually by medical experts with the consequent important investment of time and effort that this requires. We think that our methodology might also be used to generate pairs of registered images that in combination with available and valuable images registered by experts would constitute robust training samples.

Our study has several limitations. First, the central zone that surrounds the ejaculatory ducts and the anterior fibromuscular stroma of the prostate were not included in the model. However, these zones correspond to less than 25% of the volume of a normal prostate and only 10% of neoplasms arise in these zones. Second, we did not take into account how different volumes of the prostate, the TZ or the bladder influence the deformation and motion of the prostate. This could be an interesting future study for prostate biopsy and radiotherapy planification. Furthermore, the co-registration accuracy of our methodology should be validated in a phantom model or in a clinical setting. Intraprostatic fiducials for radiotherapy planning have been previously used to calculate registration errors between MRI and ultrasound in elastic fusion platforms [41]. Our biomechanical approach, although being in a pre-clinical stage, provides an extra layer of knowledge that should be taken into account by fusion imaging platforms which are the gold standard for prostate biopsies. Last, our model did not consider the deformation of the prostate by the needle which can change the geometry of the prostate during the biopsy. Our study was focused on improving the image co-registration during the planning phase of the biopsy; the additional deformation induced by needle insertion during the biopsy is indeed predicted by state-of-the-art elastic fusion platforms.

## Conclusions

In this paper we propose an alternative approach of image co-registration between MRI and ultrasound, taking into account the biomechanical properties of the pelvic tissues. Our new methodology can help predict the location of neoplasms during a prostate biopsy but further studies are needed in a clinical setting to validate our results. The proposed methodology is based on finite element simulations with an accurate and realistic 3-D geometry configuration of the pelvic region. Moreover, our methodology is completely developed on open-source software, which means that its implementation would be affordable for healthcare providers with limited budgets.

## Data Availability

The original image of the PI-RADS maps, used in the composition of Fig. 4, was extracted from the documentation for the PI-RADS v2.1 Module, which is made available by the American College of Radiology at this link: https://www.acr.org/-/media/ACR/Files/RADS/PI-RADS/PIRADS-V2-1.pdf

## Abbreviations

3-D: Three-dimensional
AI: Artificial intelligence
CT: Computerized tomography
FE: Finite element
MRI: Magnetic resonance imaging
PCa: Prostate cancer
PZ: Prostate peripheral zone
SMM: Statistical motion models
TRUS: Transrectal ultrasound
TZ: Prostate transitional zone

## References

[1] European Commission. ECIS - European Cancer Information System; 2021. Accessed 25 Jun 2021. https://ecis.jrc.ec.europa.eu/.

[2] Torre LA, Bray F, Siegel RL, Ferlay J, Lortet-Tieulent J, Jemal A (2015). Global cancer statistics, 2012. CA Cancer J Clin.

[3] Rawla P (2019). Epidemiology of prostate cancer. World J Oncol.

[4] Moe A, Hayne D (2020). Transrectal ultrasound biopsy of the prostate: does it still have a role in prostate cancer diagnosis?. Transl Androl Urol.

[5] Haskins G, Kruecker J, Kruger U, Xu S, Pinto PA, Wood BJ (2019). Learning deep similarity metric for 3D MR-TRUS image registration. Int J Comput Assist Radiol Surg.

[6] Warlick C, Futterer J, Maruf M, George AK, Rastinehad AR, Pinto PA (2019). Beyond transrectal ultrasound-guided prostate biopsies: available techniques and approaches. World J Urol.

[7] Cornud F, Brolis L, Delongchamps NB, Portalez D, Malavaud B, Renard-Penna R (2013). TRUS-MRI image registration: a paradigm shift in the diagnosis of significant prostate cancer. Abdom Imaging.

[8] Sonn GA, Natarajan S, Margolis DJA, MacAiran M, Lieu P, Huang J (2013). Targeted biopsy in the detection of prostate cancer using an office based magnetic resonance ultrasound fusion device. J Urol.

[9] Portalez D, Mozer P, Cornud F, Renard-Penna R, Misrai V, Thoulouzan M (2012). Validation of the European Society of Urogenital Radiology scoring system for prostate cancer diagnosis on multiparametric magnetic resonance imaging in a cohort of repeat biopsy patients. Eur Urol.

[10] Shen F, Shinohara K, Kumar D, Khemka A, Simoneau AR, Werahera PN (2008). Three-dimensional sonography with needle tracking: role in diagnosis and treatment of prostate cancer. J Ultrasound Med.

[11] Xu S, Kruecker J, Turkbey B, Glossop N, Singh AK, Choyke P (2008). Real-time MRI-TRUS fusion for guidance of targeted prostate biopsies. Comput Aided Surg.

[12] Penzkofer T, Tuncali K, Fedorov A, Song S-E, Tokuda J, Fennessy FM (2015). Transperineal in-bore 3-T MR imaging-guided prostate biopsy: a prospective clinical observation study. Radiology..

[13] Hale GR, Czarniecki M, Cheng A, Bloom JB, Seifabadi R, Gold SA (2018). Comparison of elastic and rigid registration during magnetic resonance imaging/ultrasound fusion-guided prostate biopsy: a multi-operator phantom study. J Urol.

[14] Delongchamps NB, Peyromaure M, Schull A, Beuvon F, Bouazza N, Flam T (2013). Prebiopsy magnetic resonance imaging and prostate cancer detection: comparison of random and targeted biopsies. J Urol.

[15] Mitsuhashi N, Fujieda K, Tamura T, Kawamoto S, Takagi T, Okubo K (2009). BodyParts3D: 3D structure database for anatomical concepts. Nucleic Acids Res.

[16] CGAL (2017). The computational geometry algorithms library.

[17] Geuzaine C, Remacle JF (2009). Gmsh: a 3-D finite element mesh generator with built-in pre- and post-processing facilities. Int J Numer Methods Eng.

[18] Electricité de France. Finite element code_aster , Analysis of Structures and Thermomechanics for Studies and Research; 1989-2017. Open-source on www.code-aster.org.

[19] Islam T, Tang S, Liverani C, Saha S, Tasciotti E, Righetti R. Non-invasive imaging of Young’s modulus and Poisson’s ratio in cancers in vivo. Sci Rep. 2020;10(1) Article number 7266:1–12. 10.1038/s41598-020-64162-6 PMC7190860 32350327

[20] Wang Y, Ni D, Qin J, Xu M, Xie X, Heng PA. Patient-specific deformation modelling via elastography: application to image-guided prostate interventions. Sci Rep. 2016;6 Article number 27386:1–10. 10.1038/srep27386 PMC4895338 27272239

[21] Krouskop TA, Wheeler TM, Kallel F, Garra BS, Hall T (1998). Elastic moduli of breast and prostate tissues under compression. Ultrason Imaging.

[22] Li C, Guan G, Zhang F, Song S, Wang RK, Huang Z (2014). Quantitative elasticity measurement of urinary bladder wall using laser-induced surface acoustic waves. Biomed Optics Express.

[23] Christensen MB, Oberg K, Wolchok JC. Tensile properties of the rectal and sigmoid colon: a comparative analysis of human and porcine tissue. SpringerPlus. 2015;4(1) Article number 142:1–10. 10.1186/s40064-015-0922-x PMC4414857 25977885

[24] Hensel JM, Ménard C, Chung PW, Milosevic MF, Kirilova A, Moseley JL (2007). Development of multiorgan finite element-based prostate deformation model enabling registration of endorectal coil magnetic resonance imaging for radiotherapy planning. Int J Radiat Oncol Biol Phys.

[25] Chai X, van Herk M, van de Kamer JB, Hulshof MCCM, Remeijer P, Lotz HT (2011). Finite element based bladder modeling for image-guided radiotherapy of bladder cancer. Med Phys.

[26] Brock KK, Ménard C, Hensel J, Jaffray DA (2006). A multi-organ biomechanical model to analyze prostate deformation due to large deformation of the rectum. Medical Imaging 2006: Physiology, Function, and Structure from Medical Images. Proceedings of the SPIE. 6143.

[27] Ramezani M, Klima S, Clerc L, de la Herverie P, Campo J, Le Joncour JB, et al. In silico pelvis and sacroiliac joint motion: refining a model of the human osteoligamentous pelvis for assessing physiological load deformation using an inverted validation approach. Biomed Res Int. 2019;2019 Article number 3973170:1–12. 10.1155/2019/3973170 PMC6343175 30729122

[28] Turkbey B, Rosenkrantz AB, Haider MA, Padhani AR, Villeirs G, Macura KJ (2019). Prostate imaging reporting and data system version 2.1: 2019 update of prostate imaging reporting and data system version 2. Eur Urol.

[29] American College of Radiology. Prostate Imaging Reporting & Data System (PI-RADS). Accessed 25 Nov 2021. https://www.acr.org/Clinical-Resources/Reporting-and-Data-Systems/PI-RADS.

[30] Creative commons attribution –NonCommercial-NoDerivatives 4.0 international license. Accessed 25 Nov 2021. https://creativecommons.org/licenses/by-nc-nd/4.0/.

[31] Igarasihi R, Koizumi N, Nishiyama Y, Tomita K, Shigenari Y, Shoji S. Sagittal alignment in an MR-TRUS fusion biopsy using only the prostate contour in the axial image. ROBOMECH J. 2020;7 Article number 4:1–7.

[32] Mohamed A, Davatzikos C, Taylor R (2002). A combined statistical and biomechanical model for estimation of intra-operative prostate deformation. Lect Notes Comput Sci.

[33] Hu Y, van den Boom R, Carter T, Taylor Z, Hawkes D, Ahmed HU (2010). A comparison of the accuracy of statistical models of prostate motion trained using data from biomechanical simulations. Prog Biophys Mol Biol.

[34] Bharatha A, Hirose M, Hata N, Warfield SK, Ferrant M, Zou KH (2001). Evaluation of three-dimensional finite element-based deformable registration of pre- and intraoperative prostate imaging. Med Phys.

[35] Marchal M, Promayon E, Troccaz J, Mendoza C, Navazo I (2006). Simulating prostate surgical procedures with a discrete soft tissue model. 3rd workshop in virtual reality interactions and physical simulations, VRIPHYS 2006.

[36] Yan D, Jaffray DA, Wong JW (1999). A model to accumulate fractionated dose in a deforming organ. Int J Radiat Oncol Biol Phys.

[37] Keros L, Bernier V, Aletti P, Marchesi V, Wolf D, Noel A (2006). Qualitative estimation of pelvic organ interactions and their consequences on prostate motion: study on a deceased person. Med Phys.

[38] Boubaker MB, Haboussi M, Ganghoffer JF, Aletti P (2015). Predictive model of the prostate motion in the context of radiotherapy: a biomechanical approach relying on urodynamic data and mechanical testing. J Mech Behav Biomed Mater.

[39] Wu G, Kim M, Wang Q, Munsell BC, Shen D (2016). Scalable high-performance image registration framework by unsupervised deep feature representations learning. IEEE Trans Biomed Eng.

[40] Checcucci E, Autorino R, Cacciamani GE, Amparore D, De Cillis S, Piana A (2020). Artificial intelligence and neural networks in urology: current clinical applications. Minerva Urol Nefrol.

[41] Moldovan P, Udrescu C, Ravier E, Souchon R, Rabilloud M, Bratan F, et al. Accuracy of elastic fusion of prostate magnetic resonance and transrectal ultrasound images under routine conditions: a prospective multi-operator study. PLoS One. 2016;11(12):e0169120:1–11. 10.1371/journal.pone.0169120 PMC5199076 28033423

